# What is Intergenerational Storytelling? Defining the Critical Issues for Aging Research in the Humanities

**DOI:** 10.1007/s10912-022-09735-4

**Published:** 2022-04-25

**Authors:** Andrea Charise, Celeste Pang, Kaamil Ali Khalfan

**Affiliations:** 1 Department of Health & Society, University of Toronto Scarborough, 1265 Military Trail, c/o Highland Hall Rm. 220, Toronto, ON M1C 1A4 Canada; 2 Graduate Department of English, University of Toronto, Toronto, Canada; 3 Department of Psychiatry, Faculty of Medicine, University of Toronto, Toronto, Canada; 4 The Health Humanities Learning Lab, University of Toronto, Toronto, Canada; 5 Research Department, Egale Canada, Toronto, Canada; 6 Institute for Life Course and Aging, University of Toronto, Toronto, Canada; 7 Faculty of Law and Odette School of Business, University of Windsor, Windsor, Canada

**Keywords:** Aging, Humanities, Arts-based research, Intergenerational, Digital storytelling, Intersectionality

## Abstract

Intergenerational storytelling (IGS) has recently emerged as an arts- and humanities-focused approach to aging research. Despite growing appeal and applications, however, IGS methods, practices, and foundational concepts remain indistinct. In response to such heterogeneity, our objective was to comprehensively describe the state of IGS in aging research and assess the critical (e.g., conceptual, ethical, and social justice) issues raised by its current practice. Six databases (PsycINFO, MEDLINE, PubMed, Scopus, AgeLine, and Sociological Abstracts) were searched using search terms relating to *age*, *intergenerational*, *story*, and *storytelling*. Peer-reviewed, English-language studies conducted with participants residing in non-clinical settings were included. One thousand one hundred six (1106) studies were initially retrieved; 70 underwent full review, and 26 fulfilled all inclusion criteria. Most studies characterized IGS as a practice involving older adults (> 50 years old) and conventionally-aged postsecondary/college students (17–19 years old). Typical methodologies included oral and, in more recent literature, digital storytelling. Critical issues included inconsistently reported participant data, vast variations in study design and methods, undefined key concepts, including *younger* vs. *older* cohorts, *generation*, *storytelling*, and whether IGS comprised an intentional *research method* or a retrospective *outcome*. While IGS holds promise as an emerging field of arts- and humanities-based aging research, current limitations include a lack of shared data profiles and comparable study designs, limited cross-cultural representation, and insufficiently intersectional analysis of widespread IGS practices. To encourage more robust standards for future study design, data collection, and researcher reflexivity, we propose seven evidence-based recommendations for evolving IGS as a humanities-based approach to research in aging and intergenerational relations.

## Background

In the field of aging research—including geriatrics, gerontology and, more recently, age studies—arts- and humanities-based (hereafter *humanities*) interventions are increasingly deployed as health- and wellness-supportive practices. Examples include cultural arts programs (Noice, Noice, and Kramer 2014), expressive and creative writing workshops (Chippendale and Bear-Lehman 2012), and participatory reading programs (Billington et al. 2013; Swinnen and de Medeiros 2018), to name only a few. Humanities-based interventions have been shown to enhance meaningful outcomes for older people, including quality of life, social engagement, and physical and mental health (Fraser et al. 2015; Young, Camic, and Tischler 2016). However, divergent perceptions of methodological rigor and the (in)appropriateness of standard outcome measures (i.e., quantitative analysis conventionally aligned with biomedical approaches) remain controversial issues facing humanities-based aging research and scholarship (Charise and Eginton 2018; de Medeiros and Basting 2014; Hanna, Noelker, and Bienvenu 2015).

One increasingly widespread example of humanities-based health research is *storytelling*, which describes the sharing of narratives that highlight the profoundly individual human experience of health, illness, and their social/cultural contexts (de Leeuw et al. 2017; Pennebaker and Seagal 1999). Health-related storytelling has been used across the life course (i.e., across youth, adult, and older-aged participants) with a vast range of health and illness-related conditions. While its purview is somewhat broader, intergenerational storytelling (hereafter IGS) may be considered an adjacent form of health-related storytelling that generally involves the oral sharing of personal and/or collective memories of lived experience, as told by one distinct generation to another. A sampling of IGS initiatives across North America and Europe might include *The Suzuki Elders Intergenerational Story Project* (Suzuki Elders, n.d.), *The Resemblage Project* (n.d.), and *The Generations Project* (n.d.).

Given its emphasis on generating and sharing narratives (Chonody and Wang 2013), intergenerational storytelling can be classified under the broader umbrella of humanities-based aging research. In referring to *generations*, we describe the tendency to demarcate population cohorts by birth date range that ascribe “shared formative context[s] . . . beliefs, [and] values” (Lyons et al. 2019, 3). In Euro-American demography, recognized cohorts include the Silent Generation (1928–1945), Baby Boomers (1946–1964), Generation X (1965–1980), Millennials (1981–1996), and Generation Z (1997–2012) (Dimock 2019). In tune with outcomes associated with intergenerational initiatives defined more broadly—including enhanced social cohesion across generations, positive health outcomes, and reduced ageism (DeSouza 2007; Stanton and Tench 2003)—IGS initiatives have been found to benefit older participants by improving health and wellbeing (Whitehouse and George 2009), increasing community involvement (Stanton and Tench 2003), and reducing isolation (Zucchero 2010). In younger people, IGS has been found to positively change attitudes about older adults (Zucchero 2010) and encourage the mutual transfer of knowledge while supporting healthy communities (Whitehouse and George 2009). Benefits for both generational cohorts include reducing generational stereotypes, promoting communication and respect, and diminishing social barriers (Hewson, Danbrook, and Sieppert 2015). For example, older and younger volunteers in *TimeSlips* (2022) both described how “their creative narrative instinct” helped “weave themselves into a multigenerational community through storytelling” (Whitehouse and George 2008, 246). The practice of sharing stories across generations thus influences individual identity and wellbeing both for the generation telling the stories and the generation listening to these stories (Merrill and Fivush 2016).

Despite such promise, however, deficient or undertheorized intergenerational interventions may facilitate misunderstanding and miscommunication between participants by perpetuating ageist stereotypes and beliefs (Kiełkiewicz-Janowiak 2012). Moreover, researchers, program coordinators, and policymakers face key implementation challenges including vastly heterogeneous intergenerational research purposes, methods, settings, target populations, outcomes, and study designs (Martins et al. 2019). While this range speaks to a promising diversity of intergenerational research approaches (including but not limited to storytelling), it presents real difficulties for generalizing about effective intergenerational measures, defining what constitutes *intergenerational* practices more broadly, and determining meaningful implementation settings.

## Study objective

What methodological approaches, practices, and defining concerns currently describe IGS as a research field? While recent work has begun to delineate the study design, interventions, and outcomes typical to intergenerational research more broadly (Martins et al. 2019), a comprehensive analysis of intergenerational storytelling as a research approach—and, crucially, the critical issues that underlie its current practice, including significant conceptual, ethical, and social justice concerns—has not yet been attempted. We define IGS as an arts- and humanities-based activity that involves sharing personal and/or collective memories of lived experience between distinct (i.e., non-adjacent) generations, using various established and emerging storytelling methods (including structured practices [e.g., reminiscence therapy, life review] and more spontaneous oral, written, performance, or digital media storytelling formats).

Our objective is to comprehensively describe the current state of IGS as an approach to aging research. We highlight common nodes of heterogeneity in published studies, identify critical issues raised by its current practice, and provide recommendations for future practices based on a methodical review of the literature. While more systematic reviews (Heyn, Meeks, and Pruchno 2019) of the field are not yet possible due to profound variations in interventions and outcomes reporting (Martins et al. 2019), this study presents a comprehensive, analytical assessment of IGS as a research field with the aim of advancing IGS as a more rigorous, critically guided approach to humanities-based research in aging and intergenerational relationships.

## Research design and methods

Published research pertaining to IGS was gathered through an extensive search of six databases (PsycINFO, MEDLINE, PubMed, Scopus, AgeLine, and Sociological Abstracts). To achieve multidisciplinary coverage, databases were selected in collaboration with a social science and humanities librarian with experience developing search strategies and literature reviews. Search term clusters related to *story* (*storytelling, stories, narrative medicine, narrative training, reflective writing, *and* creative writing*), *age* (*intergenerational, old, elderly, older adult, seniors, young, adolescents*, and* students*), and *location* (*community-dwelling*) formed the basis of the search. The search was initially conducted in January 2019 and updated (with no change in results) in June 2019.

Included studies met several criteria established before or iteratively developed throughout the search. Included studies also contained the word *storytelling*, involved distinct generational cohorts, were published or translated into English, and were published in peer-reviewed journals. No historical limitations were placed on article retrieval in order to perceive temporal shifts. Similarly, no geographical location limitations were placed on the studies so as to obtain a richer understanding of the diverse narratives of older adults within a range of communities.

In light of our focus on describing community-based, participatory intergenerational storytelling research, studies conducted with people residing in clinical settings (e.g., hospitals) or cognately age-segregated settings (e.g., assisted living, retirement communities) were excluded. Our reasoning for this was that labor-related realities of clinical spaces likely involve specific, possibly incompatible, considerations for intergenerational storytelling practices and research. Non-peer-reviewed literature, such as editorials, commentaries, and other grey literature, was also excluded.

**Fig. 1:**
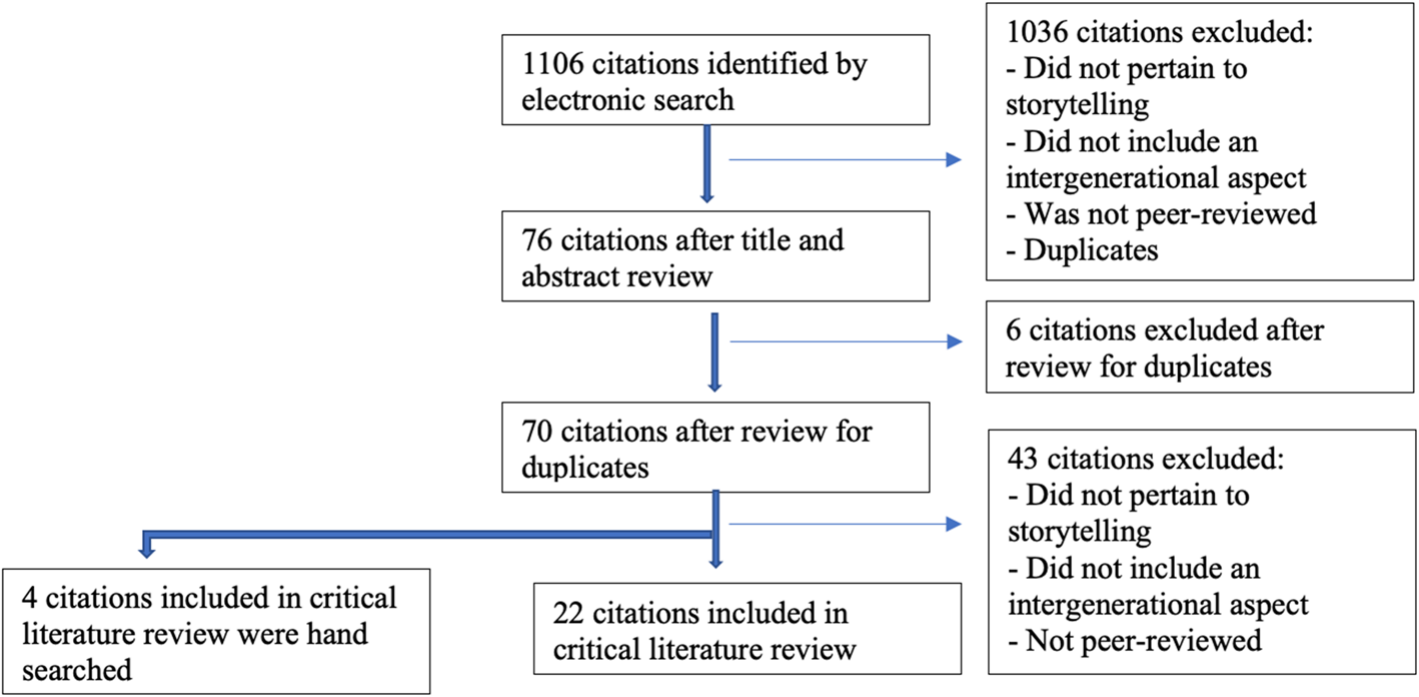
Literature search and study selection diagram

Of the 1106 initial results, titles and abstracts were independently reviewed by the authors to identify those studies eligible for further analysis (see Fig. 1). For articles where relevance could not be determined by title and abstract, the full article was independently reviewed by two authors, and differences were resolved by consensus. After removing duplicates, 70 articles were eligible for full review; of these, 22 explicitly engaged in storytelling activity between different age cohorts. Four additional articles were included after hand-searching reference lists of the 22 selected.

Analysis of included articles was based on a full review of the following data: author(s); title of study; publication year; geographical location of study; participant demographics (including reported age, sex and gender, race and ethnicity, university or college student status, and area of residence [e.g., urban or rural]); forms of storytelling media (e.g., oral, written, or digital) and storytelling approaches (e.g., life review, reminiscence, or other); research methods (qualitative, quantitative, or mixed methods); stated purpose(s) of the research; definition(s) of intergenerationality; and stated limitations and future directions.

## Results

A total of 26 eligible studies, published between 1989 and 2017, were included in this review of intergenerational storytelling research (see Table 1).

**Table 1:** Summary of included intergenerational storytelling (IGS) research studies (N = 26)

Author	Date	Title	Data Collection Method	Geographical Location
Anderson et al	2016	Translating Knowledge: Promoting Health Through Intergenerational Community	Oral (intergenerational theatre group)	Alberta, Canada
Anstadt	2009	Community Connections: An Intergenerational and Multicultural Community Group Program	Oral (*Community Connections* narrative and idea exchange)	Florida, United States
Bartlett	2005	An Intergenerational Retreat Revisited: Adolescent Girls and Older Women Share the Residual Impressions of a Single-Gender Group Experience on Female Development Four Years Later	Oral (intergenerational retreat)	United States
Bauer-Gatsos and Samatas	2017	Collecting Life Stories: A Collaboration	Oral, written (sharing and recording stories)	Illinois, United States
Chippendale and Boltz	2015	Living Legends: Effectiveness of a Program to Enhance Sense of Purpose and Meaning in life Among Community-Dwelling Older Adults	Oral, written (*Living Legends* writing program)	New York, United States
Chonody and Wang	2013	Connecting Older Adults to the Community Through Multimedia: An Intergenerational Reminiscence Program	Oral, written (story making and blog creation)	Northeastern United States
Davis	2011	Intergenerational Digital Storytelling: A Sustainable Community Initiative with Inner-City Residents	Oral, digital (interviews for digital stories)	Australia
Davis et al	2008	“I Wish We Could Get Together”: Exploring Intergenerational Play Across a Distance	Oral (storytelling interviews to create scrapbooks and magic box)	Victoria, Australia
DeSouza	2007	Intergenerational Interaction Through Reminiscence Processes	Oral (sharing life stories in class)	Brazil
Fletcher and Mullett	2016	Digital Stories as a Tool for Health Promotion and Youth Engagement	Oral, digital (knowledge-sharing sessions and digital story training)	Vancouver Island, Canada
Flottemesch	2013	Learning Through Narratives: The Impact of Digital Storytelling on Intergenerational Relationships	Oral, digital (interviews for digital stories)	Large urban area in the Midwest of the United States
Gaggioli et al	2014	Intergenerational Group Reminiscence: A Potentially Effective Intervention to Enhance Elderly Psychosocial Wellbeing and to Improve Children’s Perception of Aging	Oral, written (sharing personal memories and reporting content)	Milan, Italy
Hewson, Danbrook, and Sieppert	2015	Engaging Post-Secondary Students and Older Adults in an Intergenerational Digital Storytelling Course	Digital (digital stories)	Calgary, Alberta, Canada
Kiełkiewicz-Janowiak	2012	Narratives in Intergenerational Communication: Collaborating with the Other	Oral (interview and personal narrative sharing)	Poland
Loe	2013	The Digital Life History Project: Intergenerational Collaborative Research	Oral, digital (interviews for digital stories)	New York, United States
Momper, Dennis, and Mueller-Williams	2017	American Indian Elders Share Personal Stories of Alcohol Use with Younger Tribal Members	Oral (oral histories of harmful effects of alcohol use)	Great Lakes Indian Reservation
Nussbaum and Bettini	1994	Shared Stories of the Grandparent-Grandchild Relationship	Oral (story sharing via metaphor)	N/A
Pasupathi, Henry, and Carstensen	2002	Age and Ethnicity Differences in Storytelling to Young Children	Oral (story sharing)	San Francisco Bay Area, United States
Powers, Bailey-Hughes, and Ranft	1989	Senior Citizens as Educational Resources	Oral (story sharing in elementary classroom)	Indiana, United States
Sehrawat et al	2017	Digital Storytelling: A Tool for Social Connectedness	Digital (digital stories)	United States
Stanton and Tench	2003	Intergenerational Storyline Bringing the Generations Together in North Tyneside	Oral (creating fictional stories)	North Tyneside, United Kingdom
Tabuchi and Miura	2015	Young People’s Reactions Change Elderly People’s Generativity and Narratives: The Effect of Intergenerational Interaction on the Elderly	Oral (experience sharing)	Nishinomiya, Japan
Tabuchi and Miura	2016	Intergenerational Interactions When Transmitting Wisdom from Older to Younger Generations	Oral (experience sharing)	Nishinomiya, Japan
Thang	2006	“A Message on life to the Young”: Perceiving a Senior Volunteer Activity in Japan from an Intergenerational Perspective	Oral, experiential (museum visit and experience sharing)	Kobe, Japan
Thomson	2009	“Are We There Yet?”: Challenging Notions of Age and Aging Through Intergenerational Performance	Oral, written (theatrical performance)	United States
Zucchero	2010	Share Your Experience and I’ll Lend You My Ear: Older Adult Outcomes of an Intergenerational Service-Learning Experience	Oral (semi-structured life review)	Ohio, United States

### Participant profiles

- **Participant Demographics by Age Cohort**
  All articles reported on at least one element of demographic data for older and younger cohorts. For older participants, common demographic information included age, race/ethnicity, sex/gender, and location of residence (e.g., urban or rural).
  For younger people, demographic information generally included age, race and ethnicity, sex and gender, and university or college student status.
- **Age Identity**
  Sixteen of 26 studies (61.5%) reported the age of older participants (Anderson et al. 2016; Bartlett 2005; Chippendale and Boltz 2015; Chonody and Wang 2013; Davis 2011; Davis et al. 2008; DeSouza 2007; Hewson, Danbrook, and Sieppert 2015; Kiełkiewicz-Janowiak 2012; Loe 2013; Pasupathi, Henry, and Carstensen 2002; Sehrawat et al. 2017; Stanton and Tench 2003; Tabuchi and Miura 2015; Tabuchi and Miura 2016; Zucchero 2010). Ages ranged from 50 years old (Stanton and Tench 2003) to 89 years old (Davis et al. 2008).
  Eleven studies (42.3%) reported the age range of younger participants (Anderson et al. 2016; Bartlett 2005; Davis et al. 2008; DeSouza 2007; Fletcher and Mullett 2016; Kiełkiewicz-Janowiak 2012; Loe 2013; Pasupathi, Henry, and Carstensen 2002; Sehrawat et al. 2017; Stanton and Tench 2003; Tabuchi and Miura 2015). Ages ranged from three years old (Stanton and Tench 2003) to 62 years old (Anderson et al. 2016). One article identified younger participants only as “local teens and volunteers along with international readers” (Chonody and Wang 2013, 81), while another did not distinguish between demographic data for older and younger participants (Momper, Dennis, and Mueller-Williams 2017).
- **Race and Ethnicity**
  Eight studies (30.8%) specified the racial identity of older participants (Anderson et al. 2016; Bartlett 2005; Chonody and Wang 2013; Fletcher and Mullet 2016; Momper, Dennis, and Mueller-Williams 2017; Nussbaum and Bettini 1994; Pasupathi, Henry, and Carstensen 2002; Sehrawat et al. 2017), including three (11.5%) that consisted exclusively of racialized older participants: African American (Chonody and Wang 2013), First Nations (Fletcher and Mullet 2016), and American Indians (Momper, Dennis, and Mueller-Williams 2017).
  Eight studies (30.8%) specified the racial identity of younger participants (Anderson et al. 2016; Bartlett 2005; Fletcher and Mullet 2016; Loe 2013; Momper, Dennis, and Mueller-Williams 2017; Nussbaum and Bettini 1994; Pasupathi, Henry, and Carstensen 2002; Sehrawat et al. 2017). Two younger cohorts exclusively consisted of First Nations participants (Fletcher and Mullet 2016) or American Indians (Momper, Dennis, and Mueller-Williams 2017).
- **Sex and Gender**
  Eight studies (30.8%) reported the gender of older participants (Anderson et al. 2016; Bartlett 2005; Chonody and Wang 2013; Pasupathi, Henry, and Carstensen 2002; Sehrawat et al. 2017; Tabuchi and Miura 2016; Tabuchi and Miura 2015; Zucchero 2010), and six (23.1%) had more female than male participants (Anderson et al. 2016; Bartlett 2005; Chonody and Wang 2013; Pasupathi, Henry, and Carstensen 2002; Sehrawat et al. 2017; Zucchero 2010).
  Seven studies (26.9%) specifically reported the gender of younger participants (Anderson et al. 2016; Bartlett 2005; Pasupathi, Henry, and Carstensen 2002; Sehrawat 2017; Tabuchi and Miura 2015; Zucchero 2010), and five (19.2%) had more female than male participants (Anderson et al. 2016; Bartlett 2005; Pasupathi, Henry, and Carstensen 2002; Sehrawat et al. 2017; Zucchero 2010).
  A smaller number of studies had same-gender cohorts of either older or younger participants; two had only male participants for the older adult cohort (Tabuchi and Miura 2016; Tabuchi and Miura 2015), whereas one included only male participants in the younger participant cohort (Tabuchi and Miura 2015). Two studies reported only female participants for both older adult and younger cohorts (Bartlett 2005; Pasupathi, Henry, and Carstensen 2002), while another stated that the participant sample was predominantly female but did not distinguish the age cohort in which this existed (Chippendale and Boltz 2015).
- **Participant Location of Residence**
  Five of 26 studies (19.2%) reported on the location of residence for older participants (i.e., urban or rural areas). One study involved older adult participants from urban areas (Davis 2011), one specified that the older adult participants were from a small town (Loe 2013), and another included older adult participants and younger participants from urban areas (Anderson et al. 2016). Similarly, one study reported that both older adult participants and younger participants were drawn from rural and urban Aboriginal communities (Fletcher and Mullett 2016), whereas another described older adult participants and younger participants specifically as from the Great Lakes Indian reservation (Momper, Dennis, and Mueller-Williams 2017).
  Eleven of 26 studies (42.3%) utilized university or college students for its younger participant cohort (Anderson et al. 2016; Bauer-Gatsos and Samatas 2017; Chippendale and Boltz 2015; Davis 2011; Flottemesch 2013; Hewson, Danbrook, and Sieppert 2015; Loe 2013; Nussbaum and Bettini 1994; Tabuchi and Miura 2015; Thomson 2009; Zucchero 2010), and two of these studies indicated students were from private universities or colleges (Flottemesch 2013; Loe 2013).
- **Geographical Location of Research**
  Almost half of the included studies (12 out of 26 or 46.2%) took place in the United States (Bartlett 2005; Bauer-Gatsos and Samatas 2017; Chippendale and Boltz 2015; Chonody and Wang 2013; Flottemesch 2013; Loe 2013; Momper, Dennis, and Mueller-Williams 2017; Pasupathi, Henry, and Carstensen 2002; Powers, Bailey-Hughes, and Ranft 1989; Sehrawat et al. 2017; Thomson 2009; Zucchero 2010).
  Three studies were based in Canada (Anderson et al. 2016; Fletcher and Mullett 2016; Hewson, Danbrook, and Sieppert 2015) and three in Japan (Tabuchi and Miura 2016; Tabuchi and Miura 2015; Thang 2006). Two studies took place in Australia (Davis 2011; Davis et al. 2008), and the following countries were the location of one study each: Brazil (DeSouza 2007); Italy (Gaggioli et al. 2014); Poland (Kiełkiewicz-Janowiak 2012); and the United Kingdom (Stanton and Tench 2003). Two studies did not specify a geographical location (Anstadt 2009; Nussbaum and Bettini 1994).

### Study design and methods

- **Storytelling Media**
  Studies made use of three storytelling media: oral, written, and digital, most often in combination. Almost all studies (24 out of 26 or 92.3%) primarily utilized oral storytelling (Anderson et al. 2016; Anstadt 2009; Bartlett 2005; Bauer-Gatsos and Samatas 2017; Chippendale and Boltz 2015; Chonody and Wang 2013; Davis 2011; Davis et al. 2008; DeSouza 2007; Fletcher and Mullett 2016; Flottemesch 2013; Gaggioli et al. 2014; Kiełkiewicz-Janowiak 2012; Loe 2013; Momper, Dennis, and Mueller-Williams 2017; Nussbaum and Bettini 1994; Pasupathi, Henry, and Carstensen 2002; Powers, Bailey-Hughes, and Ranft 1989; Stanton and Tench 2003; Tabuchi and Miura 2016; Tabuchi and Miura 2015; Thang 2006; Thomson 2009; Zucchero 2010).
  Five studies (19.2%) emphasized written storytelling (Bauer-Gatsos and Samatas 2017; Chippendale and Boltz 2015; Chonody and Wang 2013; Gaggioli et al. 2014; Thomson 2009), whereas almost one-quarter (6 out of 26 or 23.1%) used digital storytelling (Davis 2011; Fletcher and Mullett 2016; Flottemesch 2013; Hewson, Danbrook, and Sieppert 2015; Loe 2013; Sehrawat et al. 2017).
- **Storytelling Methods**
  Most studies made use of life review (Butler 1963) and reminiscence (Webster, Bohlmeijer, and Westerhof 2010) as storytelling methods. Eighteen studies (69.2%) utilized life review (Anderson et al. 2016; Anstadt 2009; Bartlett 2005; Bauer-Gatsos and Samatas 2017; Chippendale and Boltz 2015; Flottemesch 2013; Hewson, Danbrook, and Sieppert 2015; Kiełkiewicz-Janowiak 2012; Loe 2013; Momper, Dennis, and Mueller-Williams 2017; Nussbaum and Bettini 1994; Powers, Bailey-Hughes, and Ranft 1989; Sehrawat et al. 2017; Tabuchi and Miura 2015; Tabuchi and Miura 2016; Thang 2006; Thomson 2009; Zucchero 2010).
  Eight studies (30.8%) employed reminiscence (Anderson et al. 2016; Anstadt 2009; Bartlett 2005; DeSouza 2007; Chonody and Wang 2013; Flottemesch 2013; Gaggioli et al. 2014; Kiełkiewicz-Janowiak 2012), while another eight (30.8%) utilized storytelling approaches not described by traditional life review or reminiscence methods, including theatre and drama (Anderson et al. 2016; Thomson 2009), scrapbooks and boxed artifacts (Davis et al. 2008), fictional storytelling and character development (Stanton and Tench 2003), memoirs (Zucchero 2010), interviews (Chonody and Wang 2013), community-foci stories (Fletcher and Mullett 2016), and metaphors (Nussbaum and Bettini 1994).
- **Research Methodologies and Analysis Approach**
  More than three-quarters of included studies (20 out of 26 or 76.9%) utilized qualitative methods in research design and analysis (Anderson et al. 2016; Anstadt 2009; Bartlett 2005; Bauer-Gatsos and Samatas 2017; Chonody and Wang 2013; Davis 2011; Davis et al. 2008; DeSouza 2007; Fletcher and Mullett 2016; Flottemesch 2013; Kiełkiewicz-Janowiak 2012; Loe 2013; Momper, Dennis, and Mueller-Williams 2017; Nussbaum and Bettini 1994; Powers, Bailey-Hughes, and Ranft 1989; Sehrawat et al. 2017; Stanton and Tench 2003; Thang 2006; Thomson 2009; Zucchero 2010).
  One study utilized a quantitative approach (Gaggioli et al. 2014), while five (19.2%) employed mixed-methods research design and analysis (Chippendale and Boltz 2015; Hewson, Danbrook, and Sieppert 2015; Pasupathi, Henry, and Carstensen 2002; Tabuchi and Miura 2016; Tabuchi and Miura 2015).

### Key terms and critical issues

- **Defining *Generations* and *Intergenerational***
  Among included studies, *intergenerationality* was inconsistently conceptualized or left incompletely defined. For example, one defined intergenerational initiatives as “programs that engage different generations, such as a younger and an older generation, in mutually beneficial planned activities” (Chippendale and Boltz 2015, 2). Another employed a similarly tautological definition provided by Ayala et al. (2007), as “programs that purposefully engage different generations (e.g., a younger and an older generation) in mutually beneficial planned activities” (Zucchero 2010, 384).
  However, in discussing an intergenerational theatre workshop exploring “the so-called ‘generation gap’,” Thomson (2009, 115) points out the potential opportunities generated by such a vague definition. Participants actively re-envisioned themselves across and through lines of generational difference in a collaborative performance: “as participants came to know one another through the sharing of personal narratives, members of the two generations began to view themselves less as ‘two generations’ and more as both unique individuals *and *as a unified whole” (Thomson 2009, 118). Here, the younger cohort was defined as those under 25 at the study’s outset and the older cohort as those over 60. While providing no concrete definition for *intergenerational* within the article, it is notable that Thomson’s findings undermine the assumption of generational *difference* as an underlying principle of intergenerational interaction.
- **Defining *Storytelling***
  Definitions of storytelling were similarly undefined or, if mentioned, inconsistently delineated. While humanities scholarship acknowledges the existence of multiple definitions of *storytelling* (Leitch 1986; Frank 2013), studies rarely distinguished between descriptive, literal impulses of communication and the more deliberately representational narrative tactics—often conflating these concepts under the general descriptor of *storytelling*. For example, one study framed the outcome of semi-structured interviews (about aging and growing old) as intergenerational communication, narrative, *and* storytelling (Kiełkiewicz-Janowiak 2012). At the time, the interviewer (in her early 20s) conducted interviews with people she defined as one or two generations older: middle-age (44–54 years old) and older (70–84 years old). In this case, the act of communicating verbally with different age cohorts was posited as constituting intergenerational storytelling.
- **Method, Outcome, or Something Else: What Is Intergenerational Storytelling?**
  Inconsistent and/or unacknowledged definitions of two key terms—*intergenerational* and *storytelling*—point to a larger critical issue across numerous studies; namely, whether IGS exists as a deliberate (i.e., a priori, hypothesis-driven) research method or approach or a retrospectively articulated outcome of some other research activity.
  For example, Momper, Dennis, and Mueller-Williams (2017) describe how IGS developed as an unplanned outcome of focus group research; in one Indigenous community, “tribal elders *unexpectedly utilized the [focus group] format as an opportunity for cross-generational storytelling* to convey their own oral histories of the harmful effects of alcohol use . . . aimed at preventing the youth from initiating drinking” (293; our emphasis). Momper, Dennis, and Mueller-Williams conclude that “the elders’ stories highlight the need to rejuvenate traditional methods of healing among AIs [American Indians] to reduce the initiation and/or harmful effects of overuse of alcohol among AI youth” (293).
  Another instance involved the citation of *generativity*—defined as a stage of psychosocial development concerned with “establishing and guiding the next generation” (Erikson 1963, 267, cited in Tabuchi and Miura 2015, 119)—as an IGS research outcome. In one Japan-based study, Tabuchi and Miura (2015) studied the effects of younger people’s reactions to older people’s sharing of wisdom through a lens of generativity; when older participants (aged 63–77 years old) expressed narratives to younger listeners, generativity was found to be promoted when the youth responded in an empathic way. In a United States-based study, Bauer-Gatsos and Samatas (2017) analyzed one IGS initiative involving university freshmen and older adult members of a church group as it promoted generativity and resiliency. In both cases, generativity was posed as a key outcome and defining attribute of IGS**.**
  However, other studies indicated how distinguishing between IGS as a deliberate research method or passive research outcome might overlook an important opportunity—a phenomenon we term the *emergent intergenerational encounter*. For example, Thang (2006) describes an initiative that emerged as the consequence of a specific event (namely, an earthquake in Japan) that spurred the creation of a volunteer narrative group (of participants in their 50s–70s) whose objective was to narrate their experiences of the event. While not initially intended as an IGS initiative or viewed by participants in these terms, Thang argues for the value of interpreting this grassroots initiative through an IGS lens to “ensure continuity and gai[n] recognition for [participants’] efforts to link the generations through narrative” (10). Thang further argues for the value of an “intergenerational eye” in assessing such research, since “charting . . . direction[s] for sustainability in the intergenerational realm” may only be “chanced upon” (20–21). Such insights signal the potential benefits of framing emergent intergenerational *encounters* as IGS after the fact, even while such strategies leave less than fully defined the parameters of what constitutes (or ought to constitute) IGS.

## Discussion and implications

Recent years have witnessed IGS become an increasingly common approach to arts- and humanities-focused aging research and practice. By focusing on the specific phenomenon of IGS, this study builds upon, while crucially expanding, a recent groundbreaking review of intergenerational programs more broadly defined (Martins et al. 2019). Alongside descriptive reporting and in keeping with IGS as a humanities-based approach to aging research, our study also integrates an evidence-based, critical perspective that highlights the affordances and limitations of current trends in IGS research.

Our results describe numerous and significant nodes of heterogeneity in IGS research ranging from participant demographics to study design and methods and key terms and concepts (see Table 2). To highlight one such inconsistency: one study’s *young* cohort included a participant who was 62 years old (Anderson et al. 2016), while another study defined *older* participants as 50 years old (Hewson, Danbrook, and Sieppert, 2015). Such variation—to do with age and aging, no less—poses a fundamental obstacle to effectively summarizing and synthesizing the evidence for IGS research (as future researchers, community program developers, and policymakers may well intend).

**Table 2:** Proposed framework of Intergenerational Storytelling (IGS) research data elements

Data Element	Example
1) Participant demographics (reported for each age cohort)﻿^a^	
Number of participants	Younger cohort (n = 20); older cohort (n = 14)
Chronological age (mean, median, range in years)	E.g., “Younger cohort ranged in age from 12–18 years old (mean 16.5, median 16). Older cohort ranged from 65–75 years old (mean 71.7, median 70).”
Gender	Offer multi-select options beyond *male*/*female* (e.g., *they*/*their* pronouns)
Racial and ethnic identity	National research entities often have best practices guidelines for collecting race and ethnicity data, which differ based on geographical context
Location of residence	Urban, rural, community-dwelling, institutionalized settings (such as university/college residence or nursing homes/other clinical settings)
2) Study design and methods	
Purpose and objectives	Include concise statement of research question, rationale, objectives/aims, and hypothesized outcomes
Geographical location of intervention site	E.g., Country, province/state, city. Recommend including profile of local and national health care access and funding (publicly funded, insurance-based, etc.)
Participant dyad structure	E.g., Postsecondary student/older adult; child/older adult; youth/E(e)lder; sex based. Also state kin-proximity and rationale for chosen dyad structure
Storytelling media	E.g., Oral, written, digital
Storytelling method	E.g., Prescribed method (life review, reminiscence) or less structured formats
IGS as research method vs retrospective outcome	Rationale to why IGS is posited as an a priori research approach, or the outcome of another activity (*emergent intergenerational encounter*)
Analysis approach and instruments	E.g., Qualitative, quantitative, mixed methods. Describe assessment tools/instruments
Intersectional analysis	E.g., Intragroup differences (Crenshaw 1991) that may exist within age cohorts introduced by factors such as race, class, and gender
Key words and concepts	E.g., Young(er), old(er), generation, age-based cohort names (for example, *youth* or *E(e)lder*), intergenerational, storytelling, intergenerational storytelling

^a^Collected data should, above all, reflect and respect the preferences of the participant community under study. Not every design and data element, particularly regarding participant demographics, may be appropriate or necessary to collect. Researchers are advised to reflect on the purpose of requesting identifying information and allow participants to self-describe and/or decline questions regarding personal identity.

In terms of study design and methods, most studies employed oral storytelling techniques. However, recent years have seen a significant uptick in the use of digital storytelling techniques, an opportunity to consider the technological form and content of intergenerational encounters catalyzed by technology use (Moreau et al. 2018). Quantitative methods were rarely employed as a means of reporting outcomes, but the continued exploration of mixed methods (blending quantitative with qualitative approaches) may present new opportunities to communicate the value of IGS to policymakers, funding bodies, and other research and practice communities.

### IGS participant dyads

Our critical analysis of key terms pertinent to IGS as a research field (including *generation*, *intergenerational*, and *storytelling*) raised some compelling implications. While the conceptual parameters of intergenerational were diffuse across studies, we identified four characteristic *dyads* that typically structure IGS study design. The first, most prevalent, was the *postsecondary* *student/older adult *dyad in IGS initiatives based at universities or colleges (Anderson et al. 2016; Bauer-Gatsos and Samatas 2017) or community-building initiatives (Anstadt 2009; Davis 2011; Flotesmech 2013; Loe 2013; Stanton and Tench 2003; Zucchero 2010). For example, Anstadt (2009) reports on the activities of a program in which international university students in the United States were paired with older adults and caretakers with one mutual aim: combatting isolation and promoting intercultural exchange through storytelling during shared meals. Possible study design and practice concerns associated with this dyad include the ethical complexities of attaching intergenerational contact to formal, mandatory, or graded pedagogical outcomes. Past research shows how socially enforced intergenerational initiatives may give rise to “do-gooding” (Dumbrell, Durst, and Diachun 2007, 950; Dumbrell et al. 2007)—namely, the perception of a generationally unidirectional savior role, with no apparent acknowledgment of reciprocal benefit from intergenerational contact—which might undermine the authenticity and purpose of IGS. Another catalyst for such concerns may arise in IGS study designs that involve technologically mediated initiatives (e.g., in digital storytelling). If younger participants are characterized, due to their age, as* de facto* technologically competent *teachers* of older participants, such initiatives may affirm rather than undermine longstanding generational stereotypes by enacting “digital ageism” (Ouellet et al. 2017, 79).

Second, a *child/older adult *dyad was evident in IGS research involving K-12 (elementary and high school) educational contexts (DeSouza 2007; Gagglioli et al. 2014; Power et al. 1989). In addition to similar concerns regarding do-gooding described above, another issue raised is the function of kin relations, proximity, and kin availability in IGS research (Pfeifer and Sussman 2014). In our study, only two articles focused on grandparents and grandchildren (Davis et al. 2008; Nussbaum and Bettini 1994), while the remainder (24 out of 26) involved non-kin-related older and younger cohorts. We see no need, at this time, to delimit IGS activities to either kin- or non-kin-related participants. However, future research may well investigate the differences that degrees of kin proximity introduce to IGS, particularly in those involving children and older relatives (as opposed to non-kin-related community members). Related to these concerns—and relevant equally to both the first and second dyads we describe—is increasing the bi- or even multi-directionality of storytelling in IGS research. In the spirit of truly *inter-*generational storytelling, researchers might more regularly solicit younger cohort perspectives and storytelling outcomes, with an eye to potentially enriched analyses consequent to disrupting the conventional old-to-young structure.

Third, a *youth/Elder* dyad was apparent in IGS initiatives involving Indigenous, Aboriginal, and/or First Nations communities (Fletcher and Mullett 2016; Momper, Dennis, and Mueller-Williams 2017). Fletcher and Mullett (2016) report on an IGS initiative in Canada facilitated to promote youth and Elder mentorship, with an aim, in part, to increase the self-esteem of youth and community connection through the creation and sharing of digital stories. In this case, youth participants ranged from 13–25 years old, while Elders were those defined as knowledge keepers within the community (not necessarily on account of age [Stiegelbauer 1996]). Momper, Dennis, and Mueller-Williams (2017) utilized focus groups in the form of talking circles as a health promotion practice against the use and abuse of alcohol; in this case, the authors used definitions provided by Indian Health Services and other tribal services (age 55 and over) to establish an Elder cohort.

Finally, *gender-based* dyads structured some IGS initiatives (Bartlett 2005; Pasupathi, Henry, and Carstensen 2002). In one notable example, Bartlett (2005) followed up with a group of younger women (aged 13–15 years old) and older women (aged 62–80 years old) who had participated in a weekend-long storytelling initiative some years earlier; not unlike the youth/Elder dyad, younger female participants were selected for having limited female role models and life challenges, while older women participants were selected to participate based on their experience of key topics (such as dating). While Bartlett (2005) notes the future research potential of IGS among boys and men (9), our search criteria did not return any such initiatives; we shall return to the critical issues raised by the third and fourth dyads presently.

### IGS study design and conceptualization

Our analysis identified key opportunities for IGS to better define its purpose, scope, and study design practices. Storytelling itself is a longstanding pillar of arts- and humanities-focused disciplines, yet IGS research occasionally conflates this practice with divergent disciplinary activities such as health communication and/or promotion. The dissemination of health-related information is, of course, a worthy and valuable goal. However, we maintain that keeping the epidemiological goals of health communication distinct from the arts- and humanities-focused disciplinary epistemology of storytelling remains an essential aspect of advocating for the arts and humanities’ value in aging research (which encourages the holistic sharing and generation of knowledge, alongside qualitative and mixed-method approaches and innovations). Positing IGS as the catalyst or outcome for health communication goals, skill-building, or other concrete objectives may well serve the interests of multiple disciplines. However, the humanities-based purpose, methods, and disciplinary impetus of IGS should, in most cases, be clearly conceptualized as a foundational element of study design.

That said, our findings also revealed reasons why researchers might feasibly cite IGS as a retrospective outcome as opposed to an a priori research approach. Based on our findings, we identified a phenomenon we call *emergent intergenerational encounters* to describe the accidental, non-purposive, or otherwise retrospectively gleaned IGS initiatives that occasioned the findings of some included studies. Emergent intergenerational encounters may take the form of grassroots, event-catalyzed, or otherwise chanced-upon IGS initiatives. We propose this concept to help researchers articulate and, in future work, better map the middle ground that exists between more conventional, hypothesis-driven approaches to IGS as a research method and comparably iterative retrospective assessments of IGS as an opportunistic outcome of intergenerational interaction.

That exception aside, however, greater lexical precision and discernment of IGS research goals should be prioritized in most cases. Nowhere was this need more apparent than in the moralizing values implied by IGS and intergenerational initiatives more broadly. Despite wide variation in the meaning of *intergenerational*, we found that its application to storytelling almost invariably constituted a *good*. One might ask: what is the problem with that? To take one example, while some participant groups were selected for a known lack of contact with other generations (e.g., Anstadt 2009; Bartlett 2005), the positing of IGS as a solution reflected two underlying assumptions: 1) that a *rift* exists between older and younger cohorts of an IGS dyad, and 2) that storytelling will inevitably do positive work in mending this rift. We argue that IGS researchers must be more reflexive, even critical, about the intensively moral values that may lie within and circulate with the use of intergenerational as a concept and the ways that storytelling posed as the *mending* of a generational rift may—or may not—be an apt assumption amongst communities studied. This suggests that IGS research must be sensitive to the consolidation of *god terms*; that is, words, phrases, and concepts that seem immune from critical apprehension on account of their apparently intrinsic good and unimpeachable value (Weaver 1953). If *intergenerational* is not yet a god term in aging research, then researchers ought to detect where, and under what conditions, claims to intergenerational *good* may exert a flattening effect in study design and analysis. Enhanced reflexivity around this issue may be especially pertinent if the field expands beyond its current geographical (United States) focus.

### IGS and/as intersectional analysis

These critical issues point to the immediate need to frame aging and intergenerationality as deeply intersectional experiences. We adopt the concept of *intersectionality*, coined by critical race theorist Kimberlé Williams Crenshaw, to describe social forces and ideological instruments that “frequently conflate or ignore intra group differences . . . such as race and class” (Crenshaw 1991, 1241). Aspects of Crenshaw’s groundbreaking work on racialized violence experienced by Black women and women of color can be brought to bear on the findings of our study. In included studies, where intersectional aspects of identity were outlined in detail (e.g., gender, age, and/or race), they rarely comprised an essential part of the analysis; nor was there evidence of identifying such nonreporting as a limitation to such research. Some attention was paid to gender as a facet of commonality, and, in multiple cases, IGS involved the gender-based pairings of women with women (as in the fourth dyad described above). However, that pairing rests on assumptions of commonality based on gendered experience, which were not questioned in researchers’ analysis.

As IGS researchers have been largely unreflexive about their parsing of gender and the underlying cisnormativity of their accounts thus far, this presents both an issue of study design as well as a conceptual problem. To further illustrate this conflation of intragroup difference, there exists little analysis on the participation of men, or questions of masculinity, in IGS research. If intergenerational pairing by gender is part of IGS programs studied, researchers should ask how and why this was the case. Doing so will better prepare researchers to integrate gender as a deliberate facet of analysis in IGS research (as in other areas of health-related research [CIHR 2019]), assess how people of different genders are involved in IGS, and consider whether there is an implied feminization of participation this field—and if so, why.

This lack of intersectional analysis in IGS extends to race, which was overwhelmingly not a facet of participant identity and demographics analysis in the included studies. Race and ethnicity for both older and younger cohorts were reported in less than one-third of published studies, suggesting the default *whiteness* of IGS. A possible exception was one study’s testing of a hypothesis about “ethnicity-related motives” in storytelling between African American and European American groups (Pasupathi, Henry, and Carstensen 2002, 610). Here, older women participants told stories to young girls in a laboratory room in a psychology department. Drawing from one theory of socioemotional selectivity theory, researchers hypothesized that African Americans would tell “more emotional stories than European Americans” and be more likely to use storytelling as a way to engage in the socialization of a nonrelative child (Pasupathi, Henry, and Carstensen 2002, 612). Beyond this hypothesis’ problematic reliance on racial stereotypes, no similarly detailed hypothesis was made for the European Americans—a trend that reflects once again what may be a conspicuous source of recruitment bias in the IGS research field at present.

Concerns may be raised with IGS studies that risk perpetuating essentialist imagery and negative stereotypes about racialized and historically marginalized participant groups in different ways, including Indigenous communities. Indeed, the question of IGS and Indigenous contexts poses a potential challenge to IGS as an emerging field of research. There is a risk, we argue, of using the language of *intergenerational storytelling* without care and reflection to describe initiatives, sites, and populations where doing so constitutes an essentially colonizing act. How does IGS articulate, for instance, with oral history storytelling and Indigenous epistemologies, pedagogies, and research approaches more generally (Iseke 2013; Hopkins 2006)? How is the field positioning, explicitly and implicitly, non-Indigenous older people as Elders (or *elders*), and what risks may exist in doing so? Is there an appropriation of space and methods or a conceptual overlap that is going unrecognized—and if so, where, and in what formats, should such recognition be made?

To the extent that aging researchers are committed to the broader task of decolonizing university-based research practices (Datta 2018; Smith 2012), we must recognize how optimistic claims of *bridging distance*—between generations, cultures, disciplines, or participant groups, to name only a few relevant to IGS research—belie the apparent neutrality of IGS as a research approach. Our findings reveal there are indeed underlying values and meanings implicit in the current state of IGS research, some of which are thoroughly imbricated in whiteness, colonization, and deeply engrained systems of social oppression. Our analysis, therefore, leads us back to our original research question: what is intergenerational storytelling? But now, it encourages us to ask more exactly: what distinguishes IGS as a research method from established, enduring cultural practices that long predate paradigms of western colonialism? IGS, we conclude, presents a provocative test case for advancing the broader decolonization of aging research. To begin, researchers might respond by acknowledging—fittingly enough—the vital old age of storytelling traditions whose value is only for some communities *emergent*.

### Limitations

To capture IGS initiatives involving community-dwelling older adults, our search terms excluded participants residing in institutionalized settings and were limited to peer-reviewed articles that fulfilled pre-established search criteria. The geographical emphasis on North American (the United States especially) and European studies may reflect our search strategy’s limitation to English-language articles. Given our interest in offering some interpretations of the numerical data reported in the included studies, it is possible that our critical and qualitative work introduced potential sources of bias. However, we maintain that the points of critique raised in this study are strongly linked to the evidence gathered by our search strategy and the descriptive data we report alongside it.

## Conclusions

As outlined in Table 2, future IGS research might consider standardizing some aspects of research and reporting, including participant demographics (e.g., age, gender, sex, race, and ethnicity), intervention site (e.g., clinical setting, community, and postsecondary institution), geographical location, keyword definitions (e.g., of cohorts, generations, intergenerational, and storytelling), kin-proximity statements, and rationale. IGS researchers are further advised to clarify the reasoning behind their choice of intergenerational dyad—including postsecondary student/older adult, child/older adult, youth/E(e)lder, and sex-based—to consider how such pairings may conflate intragroup difference or perpetuate habits of analysis insensitive to intersectional nuances of personal and cultural identity.

Based on these findings, we conclude by presenting in Table 3 several concrete recommendations for evolving intergenerational storytelling research as a meaningful, evidence-generating, and transformative approach to arts- and humanities-based aging research. To be clear, our proposals are not intended to proscribe or delimit the diversity of future approaches to IGS as a research field. Rather, we hope to initiate the development of a shared framework of conceptual and data foundations that will permit for more robust assessments—and innovative forms of implementation—of IGS research and practices.

**Table 3:** Summary of evidence-informed recommendations for Intergenerational Storytelling (IGS) research

Common IGS Research Issue	Recommendation
1) Age ranges of older and younger cohorts overlap and are inconsistently defined	There is no way to consistently segregate or uncontroversially define *older* and *younger* cohorts. Researchers should instead provide a clear rationale as to how—and why—participant dyads were chosen and defined
2) Storytelling is generally directed from older to younger cohorts, with little analysis of reverse and/or multi-directional transmission	Consider study designs and implementation sites that engage the multi-directional potential of storytelling (e.g., younger to older) alongside conventional pathways (older to younger). Researchers could also assess the appropriateness of reporting distinct cohort outcomes, and more consistently identify mutual benefits/limitations for younger and older cohorts in IGS
3) Little recognition or critique of largely western demarcations of *generation* and generational *difference*	Clarify in detail how *generation* is defined or used to structure the intergenerational contact under study, particularly in the case of communities where multi-generational households or social arrangements are typical
4) *Storytelling* as a keyword and concept is vaguely defined and occurs in a vast range of study sites	Like the wide variation and vagueness of *intergenerational*, researchers should clearly define *storytelling* as a key term drawn from the literature (humanities or otherwise). How does an IGS initiative understand *storytelling* as a *situated* practice, in light of the range of study sites (e.g., controlled environments like research institutions, university-community interactions, or community-based initiatives)?
5) A North American, primarily United States-focused, basis of knowledge	Researchers might delve deeper into the context of the United States to investigate how inequities are perpetuated and (un)accounted for in IGS programs (e.g., do IGS programs in low- vs. high-income areas influence participant outcomes?). Consider how geographical location and health system context (i.e., public vs private models) may impact purpose of IGS initiatives and outcomes
6) A lack of intersectional analysis	Where IGS participants are described in terms of race, ethnicity, or other social markers, researchers must be clear about why these categories were chosen. When reporting, demonstrate reflexivity about what generalizations are being made about populations and communities of people
7) Utilization of IGS to “rebrand” conventional health practices (e.g., health communication, public health campaigns)	While the interdisciplinary appeal of IGS should be celebrated, aging research should recognize the disciplinary origins, purpose, and value of IGS as firmly rooted in the epistemological conventions of arts and humanities. Doing so may enhance perceptions of the significance of arts- and humanities-based approaches to aging and health-related research
